# Design of Meat Product Safety Information Chain Traceability System Based on UHF RFID

**DOI:** 10.3390/s23073372

**Published:** 2023-03-23

**Authors:** Jiping Qiao, Minghui Hao, Meicen Guo

**Affiliations:** College of Electrical & Power Engineering, Taiyuan University of Technology, Taiyuan 030024, China

**Keywords:** UHF RFID, traceability system, meat products, code design, safe information chain

## Abstract

As a result of the current imperfection of the meat traceability system, there have been numerous food safety events with serious consequences. In this paper, a meat product information traceability system is designed to efficiently prevent such problems. This system develops an identification tag information reader based on ultra-high frequency (UHF) Radio Frequency Identification (RFID). It is compatible with LoRa wireless, USB serial port, RS485, and RJ45 Ethernet connection. Among them, the efficiency analysis of the Q-value algorithm finds that the recognition rate of the system reaches a maximum of about 0.367 when the number of tags n is about the frame length. The multi-tag anti-collision algorithm design based on the algorithm improves the efficiency of information collection in production and distribution links. The traceability code identification scheme is designed to effectively match various links, and the platform of system is built using LabVIEW2014 software, which has five sub-modules including user management, farm management, slaughter management, logistics management, and sales management. The system uses MySQL databases to store traceability information so that users can complete their queries by entering the traceability code on the system platform. The system not only has a low cost and a broad range of applications, but it also realizes the tracking record of meat product traceability information from breeding to selling, completes the function from information collection to information inquiry, and solves the problem of the incomplete traceability information chain. In addition, the system not only enhances the informational transparency of meat products in the product supply chain but also provides information for the regulatory authorities to effectively monitor safety.

## 1. Introduction

Numerous food quality and safety issues have arisen over the past few years, seriously hurting public confidence in the domestic food industry and raising widespread concern at all societal levels [1]. Meat products are one of the main food items that consumers are concerned about because they play a significant role at the dinner table. The economic losses brought on by historically significant food safety incidents such as lean meat extract and mad cow disease are still alarming [2]. In order to guarantee the quality and safety of meat products, it is crucial to develop a professional system of meat product traceability [3].

China has established a meat food quality and safety traceability system based on its many years of construction expertise, but it is mainly based on pilot projects for specific industries or products [4]. On the one hand, it is challenging to complete data collection and entry in an accurate and timely manner because the majority of managers and practitioners in Chinese enterprises have less education and are less proficient in using modern tools [5]. On the other hand, the standards of each link are not uniform and are not closely related, so the complete traceability of meat food from breeding to processing to circulation is not fully realized [6].

Currently, many different system architectures and software designs have been proposed in order to realize the information traceability function in the meat product supply chain. Researchers have proposed to apply RFID technology in different fields such as wine, fish, and meat in order to realize the complete traceability of the food production chain [7], a pig slaughter data collection scheme to effectively address the issues of harsh environmental conditions and difficulty in the pig slaughter processing process [8], a Petri net-based and UML-based modeling approach to identify sheep meat traceability information and processes [9], and the use of DNA technology for traceability systems to prove the origin of Swiss meat [10]. It is clear that more researchers are focusing on the development of food traceability systems, but they are concentrating more on the technology and method research for a specific object or link and less on the hardware, the connection of various links, and the platform software to realize the whole traceability system.

The Internet of Things (IoT) technologies are immensely helpful in the food supply chain. Nowadays, the rapid rise of the technology and the countless application studies in the traceability system have further promoted the establishment of the food traceability system [11,12,13]. One of the key components of IoT is RFID technology [14,15], which enable remote data storage and retrieval from electronic tags without the need for mechanical contact, enabling the monitoring and tracking of objects after they have been identified. Its main benefit is that the tags can be read through snow, fog, and other harsh environments, and the recognition speed is very quick. RFID technology areas include material handling, supply chain management, and manufacturing, covering a wide range of applications [16]. In general, an RFID system consists of three main components: an RF tag, a reader, and a computer [17]. According to the working frequency band, the system can be divided into low-frequency, high-frequency, ultra-high-frequency (UHF) and microwave systems [18]. In comparison to low- and high-frequency RFID, UHF band RFID technology works more effectively and can recognize more tags over greater distances with a faster rate of recognition. It has currently become a hotspot for research and development in the field of radio frequency identification technology on a global scale as the application field of UHF RFID technology is expanding [19].

The system comprehensive application of UHF RFID technology, wireless communication technology, database storage technology, and other cutting-edge science and technology. It is of great value and significance to realize the collection and storage of information related to the stages of meat breeding, slaughtering and processing, logistics and transportation, and product sales, and to build a safety information chain traceability system. It can assist businesses not only in carrying out precise management and ensuring the safety of meat production and supply, but also in realizing the traceability of the entire product production and sale, protecting consumer needs. The main contribution of this paper is the development of a low-cost, practical UHF RFID reader for accurate collection of key information about different batches in different links. The second improvement is to create an information marking scheme in the meat product distribution process for effective matching in different links.

## 2. Materials and Methods

### 2.1. Design of System Framework

**Figure 1:**
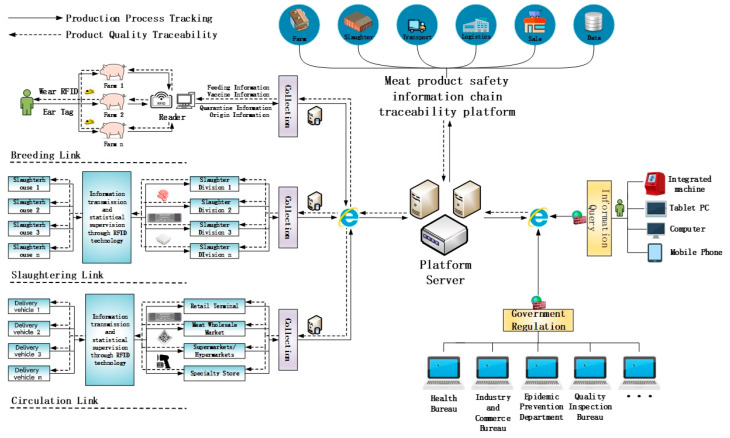
The business process of traceability system.

The traceability management of the meat safety information chain is a challenging and time-consuming task. It incorporates numerous components. The traceability platform mainly includes several parts such as breeding management, slaughter management, logistics management, sales management, and data management. The main framework of the system and business processes are shown in Figure 1.

The proposed traceability platform mainly records information on breeding, slaughtering, and circulation links. In the breeding link, the breeder attaches the RFID ear tag with the EPC data to the livestock and binds it. A reader then reads the information from the ear tag to gather details about the type of livestock, feed, quarantine, and other information until it is sold. The electronic ear tag needs to be converted to EPC paper labels in the slaughter link because livestock is slaughtered, processed, separated, and packed. It is no longer appropriate as an information carrier. Similarly, the slaughter link uses the RFID reader to read the information from tags and bind them at each step in livestock processing. The distribution link maintains the same labeling scheme, it continues to use the paper label after slaughter to carry logistical and transportation information in the distribution link. However, there are many information tracing methods that can be considered in the sales process depending on the actual situation, such as paper labels, two-dimensional codes, and bar codes. Eventually, the data gathered by each link is uploaded to the local database and sent to the central platform database via the Internet. Consumers and government authorities have a variety of options for Internet connection to the platform server and request the necessary traceability data.

### 2.2. Hardware Design

This paper proposes an EPC C1 G2 protocol supported design for the UHF RFID reader. The primary controller of the hardware used a STM32F103RET6 microprocessor (STMicroelectronics, Shanghai, China), and the MagicRF M100 chip (MagicRF Co., Ltd., Jiangyin, China) serves as the core of the RF transceiver component. Additionally, an external power amplifier circuit was designed to increase the recognition distance, in consideration of the two crucial elements of output power and efficiency. Some of the key metrics specifically achieved are as follows:

- Supported Protocols of Systems: Supports the requirements of the EPC Class-1 Generation-2 protocol. The reader could realize many functions such as tag selection, data access, and inactivation of EPC;
- Range of operating frequency: the proposed system is in UHF (840–960 MHz) [20];
- Power supply: the functional modules were all DC supplied by a 12 V power adapter, and other circuits were supplied by 5 V and 3.3 V DC power transformed from 12 V power through a power conversion chip;
- Radiofrequency power: a maximum RF output power of 26 dBm was designed while the system achieved a step of 1.5 dB adjustable transmit power;
- Communication method: it is compatible with many communication protocols such as USB, LoRa wireless communication, 485 connection, and RJ45 network connection.

**Figure 2:**
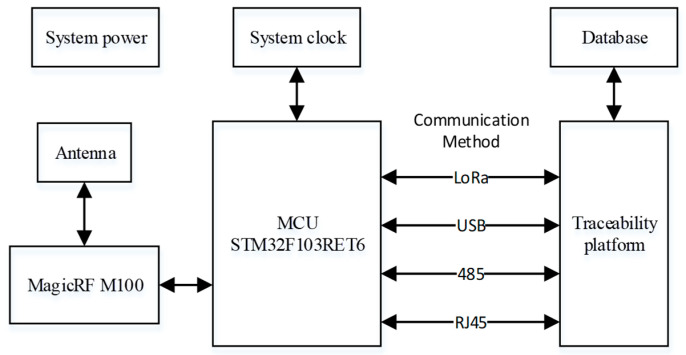
Hardware circuit design.

The design layout of the reader system is shown in Figure 2. It primarily consists of four modules, including an RF transceiver circuit, the main control circuit for the STM32F103RET6 microcontroller, a peripheral connection circuit, and a traceability platform component. At the same time, the reader and traceability platform support a variety of communication types, so that the actual application is more extensive.

#### 2.2.1. Design of Power Module

**Figure 3:**
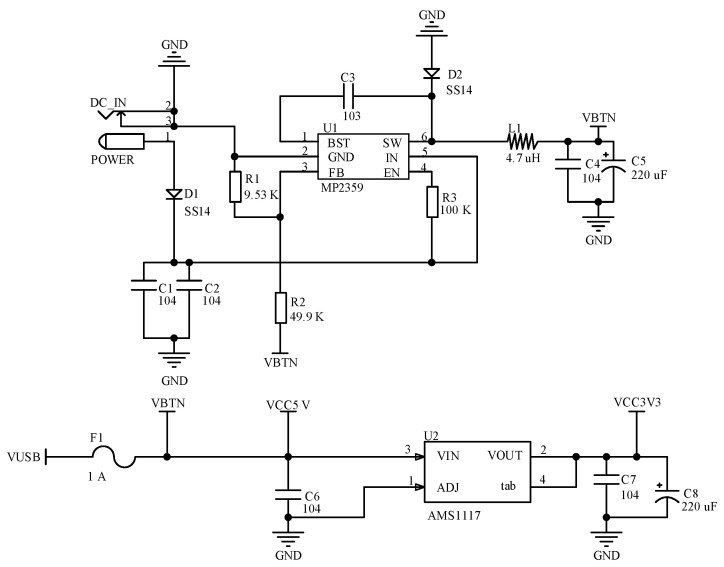
Power supply circuit.

The MP2359 DC buck chip was chosen for the design of the 5 V supply voltage generation circuit in the power supply section. It has an integrated DC/DC converter and the maximum output current for the chip is 1.2 A. The AMS117 voltage regulator chip was used to convert 5 V to 3.3 V. The circuit input is 12 V DC power adapter for the system. It is shown in Figure 3, where the VBTN is 5 V voltage.

#### 2.2.2. Design of RF Module

The MagicRF M100 chip in the system uses a 32-Lead QFN package which is the smallest package size of any modern international RF chip. In addition, the chip has an integrated single-ended output power amplifier of up to 4 dBm. When working at maximum power, the internal current of the chip is only 80 mA. The sensitivity of the chip is around −69 dBm at −10 dBm local blocking. It can detect much fainter signals [21].

**Figure 4:**
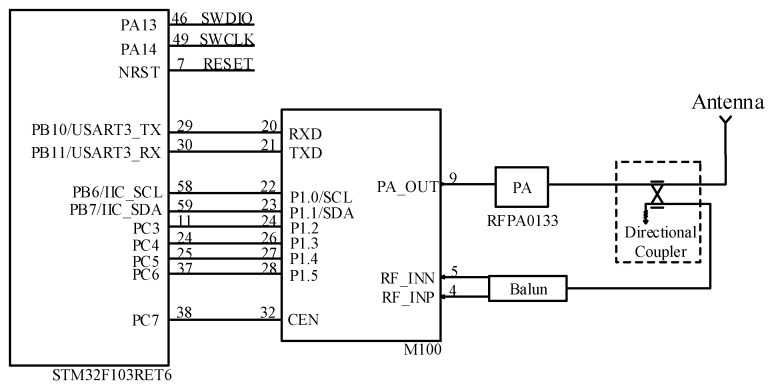
RF interface circuit.

**Figure 5:**
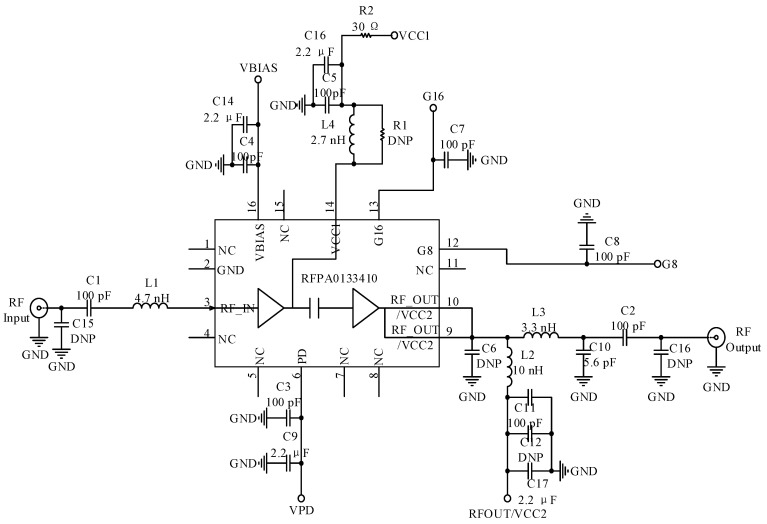
Power amplifier circuit.

The hardware interface circuit is shown in Figure 4. The key auxiliary circuits of the RF section have four parts, including balanced and unbalanced converters, external power amplifier circuits, filters, and directional couplers. The RFPA0133410 was chosen as the external power amplifier chip with a gain of roughly 32 dB. The DC0710J5020AHF directional coupler chip was chosen to realize the RF signal between sending and receiving. The circuit is shown in Figure 5.

#### 2.2.3. Design of Communication Module

Due to the complexity of the actual application and the communication circuit need to adapt to a variety of hardware receiving interfaces, this reader has four different types of communication including LoRa wireless, USB serial port, RS485, and RJ45 Ethernet.

**Figure 6:**
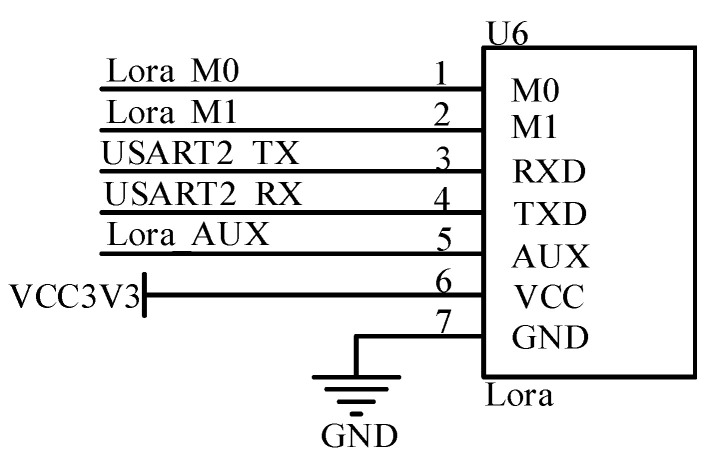
Lora communication circuit.

The LoRa wireless communication circuit utilized the wireless serial LoRa module of the SX1278 chip (Semtech, South California, CA, USA) for circuit design. It is based on the consideration of a moderate amount of data as well as a closer read-write distance between the server and the meat products in the circulation link. The module uses LoRa spread spectrum technology. It significantly enhances anti-interference and sensitivity, and the operating frequency band of 410–441 MHz extends the range of communication. The circuit is shown in Figure 6.

**Figure 7:**
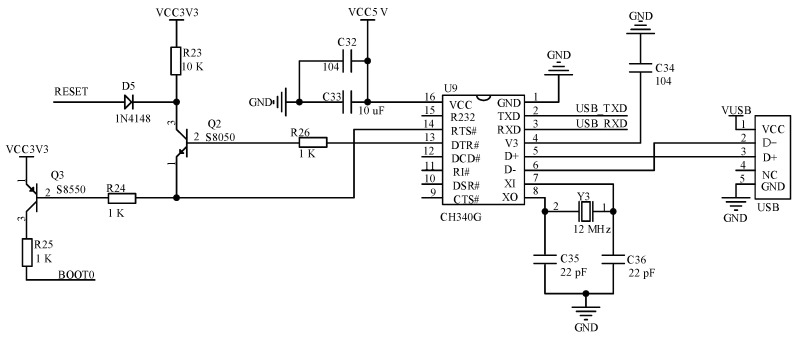
USB communication circuit.

The USB serial communication circuit supplies a 5 V DC power supply through the USB interface. It also used the CH340G chip to implement level conversion for communication between the MCU and computer. The circuit can download and debug software programs as well as perform serial data transfer. The USB serial communication circuit is shown in Figure 7.

**Figure 8:**
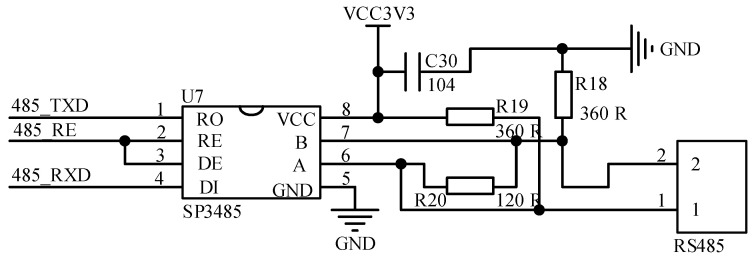
485 communication circuit.

This paper used SP3485 chip to implement the TTL level to 485-level communication. The chip is a low-power half-duplex transceiver that operates at +3.3 V. It can perform remote data acquisition at rates of up to 10 Mbps (with load). The SP3485 chip connects to the 485 lines by using the A and B bus interfaces. The communication circuit is shown in Figure 8. RO is the receive output, DI is the transmit data income, RE is the receive enable signal (active low), and DE is the transmit enable signal (active high). In this paper, a 120-ohm matching resistor is connected between interfaces 1 and 2 in order to remove noise interference. The implementation of 485 communication in software is actually the same as serial communication. The sender and receiver did communicate data in accordance with the serial port standard. When doing one-to-many communication, the software generates a certain format for transferring data frames, and the device address number needs to be edited. The receiver assesses the validity of the data based on the address number.

**Figure 9:**
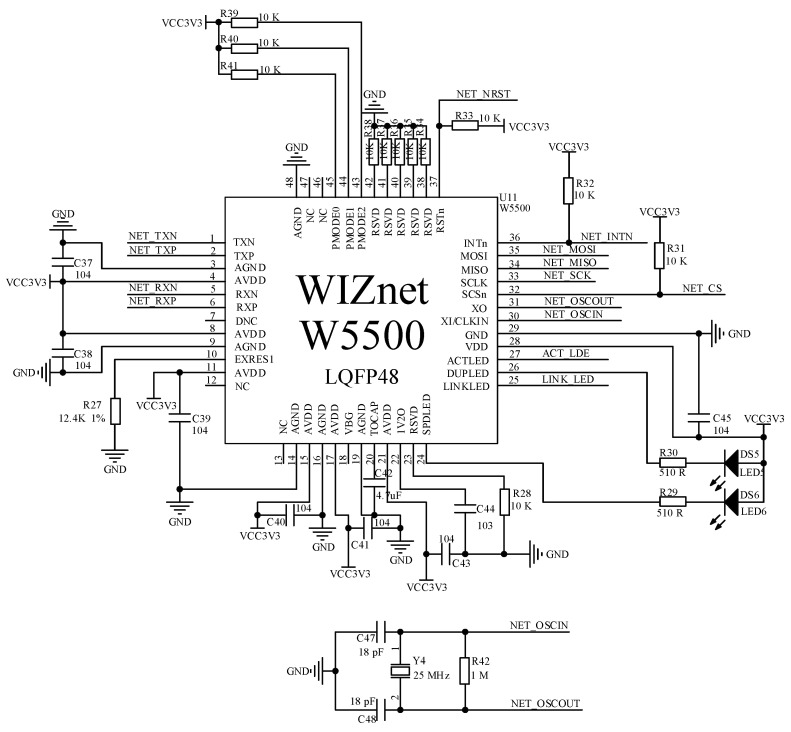
RJ45 communication circuit.

In RJ45 network port communication, this paper chooses the more advantageous W5500 chip as the driver chip for RJ45 network port communication by comparing hardware and the TCP/IP protocol stack. The chip is packaged in a 48-pin LQFP lead-free package and has advantages such as high performance and value for money. In addition, using the W5500 chip for communication in microcontroller software development leads to less code and less flash storage space required on the microcontroller. The Ethernet driver circuit of the reader system is shown in Figure 9.

### 2.3. Software Design

#### 2.3.1. Design of Reader Configuration Platform

**Figure 10:**
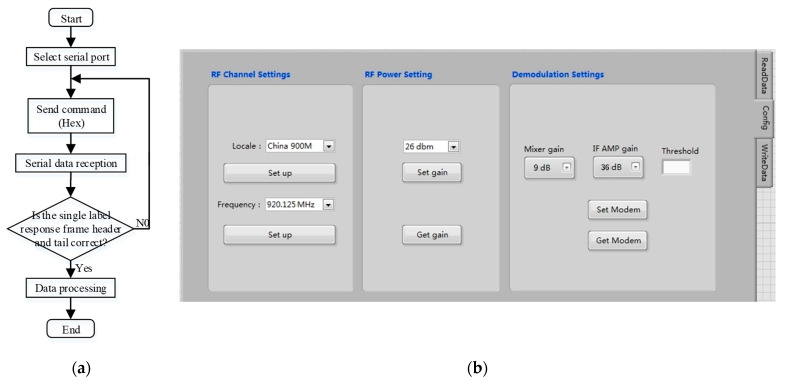
(**a**) Communication process of reader configuration platform; (**b**) Interface of reader configuration platform.

In this system, commands and data are sent and received by the hardware system through the USB interface by using the VISA serial module in LabVIEW. It can also realize the configuration function of the working frequency and gain of the reader. The reader configuration platform is shown in Figure 10.

#### 2.3.2. Design of Business Function Module

**Figure 11:**
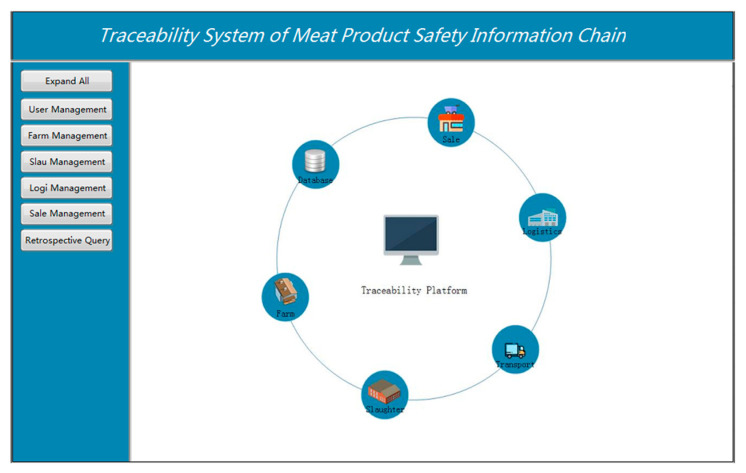
System platform.

The meat supply chain process involves numerous links, including farming, slaughtering and processing, warehousing and logistics, distribution, and retail. It makes it more difficult to supervise the quality and safety of food. This paper divides the supply chain into several modules such as breeding, slaughtering, logistics, and sales by analyzing the production and operation processes of each link. It is important to record the key traceability information of each module one by one on the system platform. It is to ensure the integrity of the information chain in the circulation process. In this paper, the business function module of the meat safety information chain traceability platform includes user management and login, breeding management, slaughter management, logistics management, and sales management sub-modules. The main interface of the traceability platform is shown in Figure 11.

The livestock breeding stage of the supply chain has the longest time cycle, and it serves as the primary point of collection and control for all traceability information. Therefore, there are numerous traceability indicators that need to be recorded in the link. This paper defines two tables in the MySQL database for the agricultural link. On the one hand, the farming enterprise must keep track of important information such as the name, address, phone number, date of registration, and capital, among others. On the other hand, the type of livestock, entry time, ID number, breeder, feed information, vaccine information, quarantine information, and slaughter time were made into records and bound with the farm name at the same time. After entering the fence, the livestock will wear RFID electronic ear tags, and this paper considered livestock from the same breeding house as the same batch. The specific implementation process can be described as follows: The remote computer accesses the reader mounted on the breeding house when recording crucial node information such as feed, epidemic prevention, and livestock medication. Multiple livestock ID numbers are transmitted back by the reader for the batch. The computer binds the breeding information to these numbers and uploads the data to the breeding base data server.

In the slaughter management stage, it is crucial to ensure the consistency of traceability information, because the link transformed the RFID ear tag in the breeding stage into a paper tag. It also served as the first transformation of the identification carrier method. After that, the operation data of various processing nodes was linked to the ID of the paper tag for database storage. This system defines two tables in the database for key information. On the one hand, the slaughterhouse name, address, contact number, capital, and registration time were recorded. On the other hand, key node information was recorded such as pre-slaughter quarantine, slaughter transfer time, livestock ear tag ID number, pre-slaughter quarantine, slaughterer person, meat tag ID number, and slaughter transfer time. For the qualified meat bound RFID traceability tag, which is the meat food tag ID number. The computer will bind the traceability tag with the ID number of the livestock and store it in the database. After that, the food code was bound to the selling unit by using the radio frequency identification channel. The related data were uploaded to the database of the traceability system platform for storage and future monitoring by consumers and government agencies.

The logistics management link is critical to keep track of the upper and lower node businesses in the supply chain. It ensures the logistics and transportation environment to achieve the quality and safety of meat products. In this link, this system primarily records two aspects. It keeps a record of important details, including the name, address, phone number, date of registration, and capital of the logistics company. It also records important node details such as logistics transfer time, product EPC code, transit temperature, and logistics transfer time.

**Figure 12:**
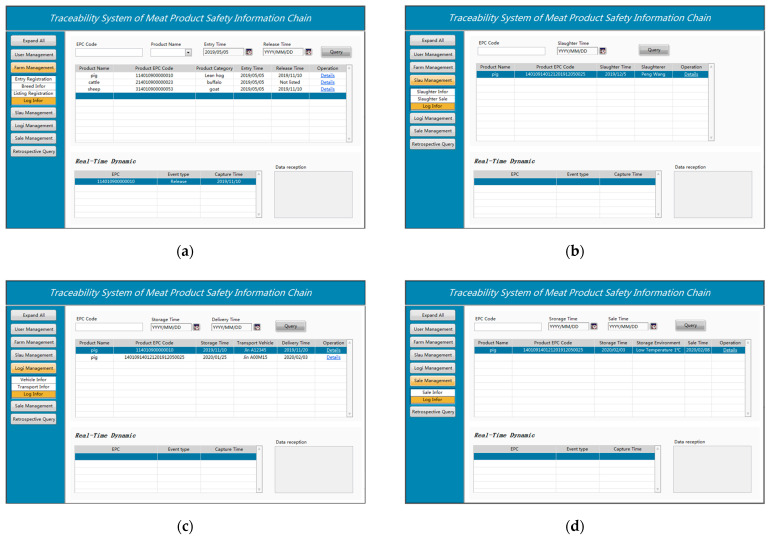
(**a**) Log query of farm management; (**b**) Log query of slaughter management; (**c**) Log query of Logistic management; (**d**) Log query of sale management.

Generally speaking, supermarkets, hypermarkets, specialty shops, and other types of businesses are the main sales terminals for meat. In this process, the original RFID paper tags can be used to preserve traceability data for the split and refrigerated meat products in the warehouse. RFID tags can be turned into inexpensive QR codes or barcode tags for the portion of on-site cutting and selling to give consumers retail traceability credentials, and relevant data can be uploaded to the platform data center. The sales management sub-module mainly consists of two parts: on the one hand, it records key data such as the name, address, phone number, and capital of the sales company; on the other, it records key node data such as the time of the sales transfer, ID number of the meat product, preservation temperature, and QR code or barcode information. The query interface of each section is shown in Figure 12.

### 2.4. Design of Multi-Label Anti-Collision Algorithm

**Figure 13:**
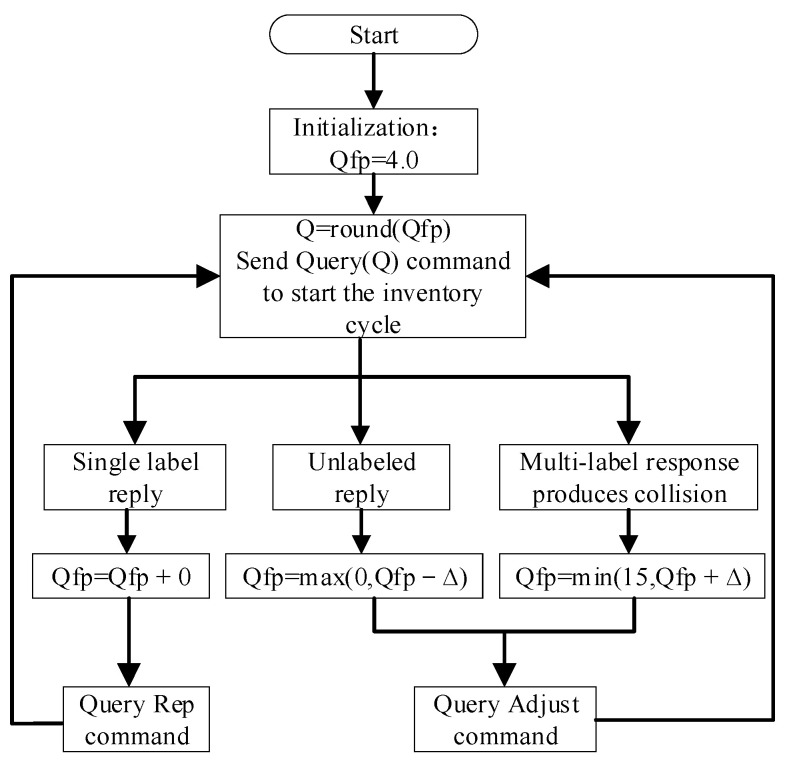
Q algorithm program flowchart.

This paper chose Aloha algorithm for design research considering that the UHF RFID system reader hardware cannot implement the detection of specific collision bits. When reading multiple labels using the Q value method, the reader sends out a command, but when numerous labels respond at once, the reader is unable to operate the label normally. This is referred to as “label collision”. Therefore, multi-label reading is necessary to solve the label anti-collision problem. The time slot Q method offered by the EPC C1 G2 protocol is used in the tag anti-collision processing algorithm module developed in this research. The Q algorithm is an anti-collision processing algorithm of the Aloha type. It is independent of the sequence number and makes use of dynamic frame time slots. It is built on the concepts of probability and slotting [22,23]. As shown in Figure 13, the specific flows of the Q algorithm are as follows:

1. First, the reader initializes a floating-point number Qfp with a value of 4.0, then rounds Qfp, and assigns the value to Q. Subsequently, a Query instruction is sent that includes Q. If the tag receives the Query instruction, it generates a random number in the range 0 to $2^{Q}$−1 and records the number in the corresponding time slot counter. At this moment, the tag with a slot value of 0 will respond immediately, and the other tags will not respond;
2. In the event that there is just one tag response, the tag will send its RN16 to the reader to establish communication and reflect the EPC to the reader, then exit after the communication ends. Subsequently, the reader sends the command “Query Rep” to other tags, and each tag that receives the command deducts 1 from the value recorded in the time slot counter;
3. The reader uses steps to adjust the Qfp value when there are no or numerous tag responses. Qfp minus the constant ∆ but must be ensured to have a minimum value of 0 for the former (empty time slot), Qfp plus ∆ but must be ensured to have a maximum value of 15 for the latter (conflicting time slot), where ∆ is a constant and takes a value in the range 0.1 to 0.5. Based on the new Q = round (Qfp) value after adjustment, the reader decides whether to continue using the current frame or begin a new frame using the Query Adjust command.

The performance of passive UHF RFID systems is primarily influenced by the tag recognition rate. The tag recognition rate is calculated as the number of time slots with just one tag selected divided by the total number of time slots in the system. The total number of time slots is calculated by adding the sum of successful time slots with one tag, idle time slots without a tag, and collision time slots with multiple tag selections. Therefore, the label recognition rate S is:

(1) $$S=\frac{N_{s}}{N_{s}+N_{c}+N_{e}}$$

where $N_{s}$ is success time slot, $N_{c}$ is collision time slot, and $N_{e}$ is idle time slot.

When using anti-collision algorithms of the Aloha type, the correlation between the quantity of time slots and the quantity of tags is typically used to determine system efficiency. The efficiency of the system is low when there are few tags and many free time slots. The relevant conclusion demonstrates that the throughput rate can reach its maximum when the number of tags and time slots are equal, and the system efficiency is at its highest at the moment. The relevant proofs are shown below:

Assume the read-write around the waiting recognition electronic label number is n and the frame length is L. Because all the labels in the selection time slot are independent and do not produce influence, the read-write method follows the binomial distribution law when choosing any integer at random from the [0, L] range. As a result, there are r tags at the same time, corresponding to the ith time slot probability P as:

(2) $$P\left(m=r\right)=C_{n}^{r}\cdot {\left(\frac{1}{L}\right)}^{r}\cdot {(1-\frac{1}{L})}^{n-r}$$

When r = 1, only one electronic tag produces a response, indicating that the ith time slot is a successful time slot, at which time the probability P is:

(3) $$P\left(m=r=1\right)=C_{n}^{1}\cdot \left(\frac{1}{L}\right)\cdot {\left(1-\frac{1}{L}\right)}^{n-1}$$

The expectation E of having a successful time slot in a frame is:

(4) $$E\left(m=1\right)={L\cdot C}_{n}^{1}\cdot \left(\frac{1}{L}\right)\cdot {\left(1-\frac{1}{L}\right)}^{n-1}$$

The throughput rate η of the system is:

(5) $$\eta =\frac{E\left(m=1\right)}{L}=C_{n}^{1}\cdot \left(\frac{1}{L}\right)\cdot {\left(1-\frac{1}{L}\right)}^{n-1}=\left.\frac{n}{L}\right.\cdot {\left(1-\frac{1}{L}\right)}^{n-1}$$

Derivative of n to find its poles:

(6) $$L=\frac{1}{1-e^{-\frac{1}{n}}}\approx \frac{1+\frac{1}{n}}{1+\left(\frac{1}{n}-1\right)}=n-1$$

The analysis shows that the recognition rate of the system reaches the maximum when the number of tags n is about the frame length.

(7) $$S_{\max}=\left.\frac{n}{L}\right.\cdot {\left(1-\frac{1}{L}\right)}^{n-1}\approx \left.\frac{n}{L}\right.\cdot e^{-\frac{n}{L}}\approx 0.367$$

**Figure 14:**
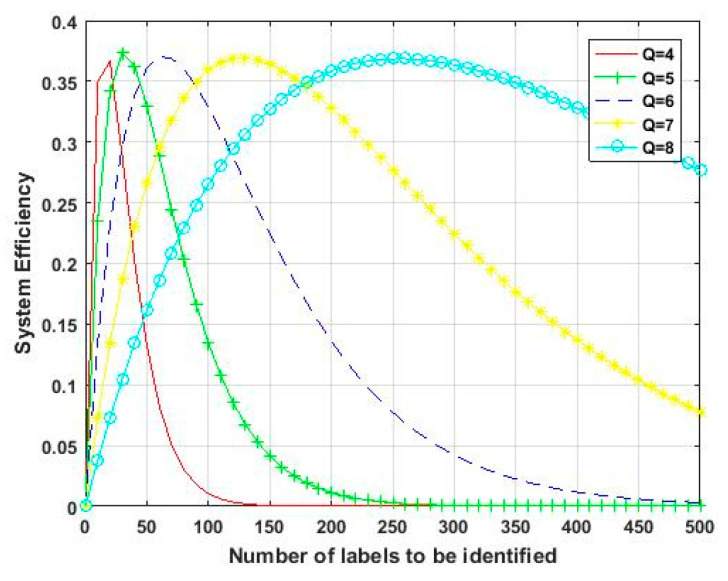
System efficiency for different Q values.

The corresponding system efficiencies at different Q values are shown in Figure 14.

The Q-adjustment algorithm is the frame length adjustment technique that is frequently employed in current UHF RFID systems because of its high recognition efficiency and the number of correctly recognized tags per unit of time.

### 2.5. Design of Meat Product Code Marking Scheme

RFID ear tags and labels used in this research have a storage capacity of 96 bits for the EPC code, which is the ID number of ear tag or label and is similar to an ID card. It can be set up with a large number of identifiers, which makes sure that each individual animal or product has a unique ID as much as possible. In addition, ear tags and labels can be read and written multiple times, and even encrypted if necessary.

This paper applies the individual ear tag labeling technique during the breeding stage of livestock. According to the relevant requirements of the Ministry of Agriculture, the type of livestock and poultry, the county and municipal area code, and the identification sequence number make up the differentiation code for livestock. Among them, 1, 2, and 3 are used to represent the categories of pigs, cattle, and sheep, respectively. This specific coding is as follows: the first bit indicates the livestock type code, the middle six bits indicate the county and municipal administrative area code of the region in which the farm is located, and the final eight bits indicate the entry sequence identification number, which is made up of a total of fifteen bits.

This paper converts the ear tag into an electronic tag for the slaughter and processing link and specifies in the design analysis that the marking scheme used is the same after the meat products are slaughtered and leave the factory until the sales link. The difference is that the system uses RFID paper tags before the slaughter reaches the sales link, while a barcode or 2D code printed by a special printer can be used in the sales link. This study also designs a 24-digit marking scheme for the meat product tag in order to completely use the EPC storage capacity and bind the information related to the farm and the slaughterhouse. The first 6 digits are the middle 6-digit administrative area code used to associate livestock in the breeding process; the next 6 digits are the administrative area code of the slaughterhouse; and the next 12 digits represent the date of slaughter of livestock (8 digits) and the slaughter order number (4 digits) of the slaughterhouse livestock located in the batch. After calculation, the 4-digit batch sequence numbers can be specified in the quantity range of 0 to 9999, which can meet different scales for slaughter marking requirements.

## 3. Results and Discussions

### 3.1. Reader System Test

**Figure 15:**
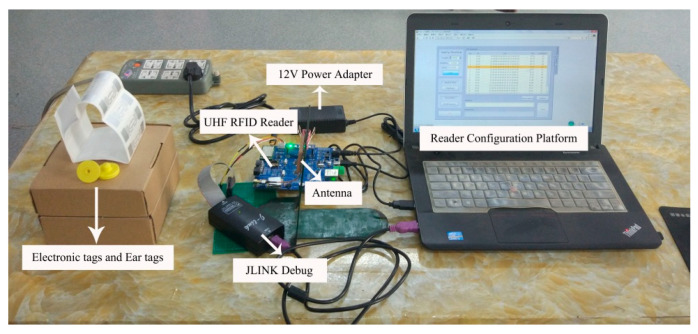
System test platform.

The test platform for this system is shown in Figure 15. The test electronic tags and ear tag chips come from the Alien Higgs-3 series chips, which support the ISO/IEC 18000-6C standard protocol and EPC Gen2 (V1.2.0) [24,25]. The chip has 800-bit non-volatile memory, which is divided into four blocks: User, TID, EPC, and Reserved. The User area is the user area and stores user-defined data; the TID area stores the tag identification number, each of which is unique; the EPC area stores EPC, EPC-PC, and other data; and the Reserved area is the reserved area and stores access password and kill password data. In addition, the data can be written up to 100,000 times with good performance. When communicating with the traceability platform, the reader supports LoRa wireless communication, USB serial port, RS485, and RJ45 Ethernet interface, which makes it more convenient for the hardware system to communicate with the platform. In comparison to barcode technology, RFID technology has remote data storage and retrieval capabilities, as well as the ability to read the data content of multiple tags at once. Additionally, the system has the ability to use encryption to safeguard the coded identification information, further enhancing its reliability.

In order to activate the internal circuit of tags and make it work, readers of tags must supply 10 to 30 uW of energy, and more energy is needed to rewrite the internal data of tags. The rectifier circuit supplies tag energy, but because some devices in the circuit require start-up voltage, there will be some energy loss, and only 30% of the energy is actually used. The antenna needs to obtain between 30 and 100 uW of power in order to supply the energy needed for the internal chip to work properly. As shown below:

(8) $$P_{RX_{\min}}=3\times 10^{-5}\;W,\;P_{RX_{\max}}=1\times 10^{-4}\;W$$

According to Friis formula:

(9) $$P_{\mathrm{RX}}=P_{\mathrm{TX}}G_{\mathrm{TX}}G_{\mathrm{RX}}(\frac{\lambda }{4\pi R}{)}^{2}$$

Approximate transmission distance:

(10) $$R=\frac{\lambda }{4\pi }\sqrt{\frac{P_{\mathrm{TX}}G_{\mathrm{TX}}G_{\mathrm{RX}}}{P_{\mathrm{RX}}}}$$

where $P_{\mathrm{TX}}$ and $G_{\mathrm{TX}}$ are the transmit power and antenna gain of the system, $P_{\mathrm{RX}}$ and $G_{\mathrm{RX}}$ are the threshold power and antenna gain of the tag chip, respectively.

After testing, the maximum recognition distance can reach 5 m to meet the actual demand.

**Figure 16:**
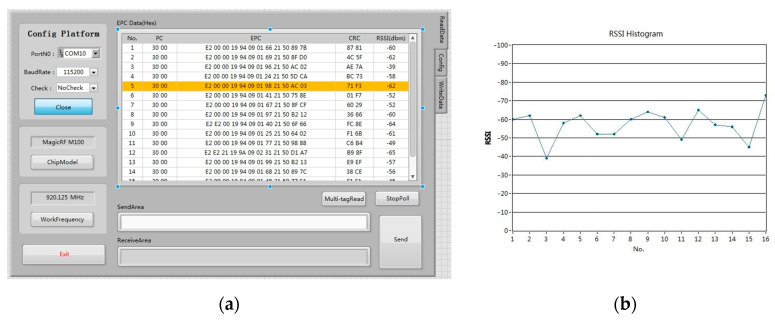
Test results: (**a**) Network port label data; (**b**) RSSI data analysis.

The platform interface is shown in Figure 16a. The number of the connected serial port is COM10, the baud rate is 115,200, and check bits are shown in the top left corner. The stop bits and data both have default values. Below is the current chip model of the reader and the current frequency band acquisition. The hardware reads all of the tag information from the right side. The EPC table, from left to right, includes read tag serial number, the PC code indicates the physical characteristics of the tag, the EPC code indicates the information on the tag, the CRC code is used to identify errors, and the RSSI value indicates the received signal strength. The above PC, EPC, and CRC codes are hexadecimal representations. As shown in the picture, the obtained chip model is MagicRF M100, and the current operating band is 920.125 MHz. The reader connects to the computer through serial port 10 successfully. The result of reading multiple tags is shown in the table content in the figure. The serial number is 5, the PC code is 3000, the EPC code is E20000199409016921508FD0, the CRC check code is 4C5F, and the RSSI value for the tag information is −57 dBm. The EPC code is the tag ID, which is 96 bits of data translated into hexadecimal, which is 24-bit data. Figure 16b shows the RSSI value, which indicates the signal strength of several tags. The RSSI value basically remains between −40 dBm and −70 dBm when the system recognizes multiple tags, indicating that the system link quality is high and operation is stable in practical applications. After testing, the read-write maximum gain of 26 dBm, can achieve adjustable transmit power in steps of 1.5 dB.

The low cost of the system is one of its significant advantages. The STM32F103RET6 microcontroller was chosen for the system because it offers a lower-cost platform based on the Cortex-M3 core, fewer pins, and lower system power consumption. The main RF chips on the market are PR9000, AS3992, and R2000. Although these chips perform well, they have a number of disadvantages, such as high prices, the need for imports, and difficulty in purchase. This system uses MagicRF M100 chip, which has many advantages, including low cost, small size, and low power consumption. ZigBee, 4G, and LoRa are currently popular wireless communication technologies. ZigBee, although low-cost, is a short-range wireless communication technology. 4G communication technology, the cost is relatively high. The LoRa technology used in this system is a long-range low-power data transmission technology that achieves the unity of low-power, low-cost, and long-range. The reader achieves high performance at low cost in terms of controller, chip selection, and communication technology.

### 3.2. Traceability Platform Test

**Figure 17:**
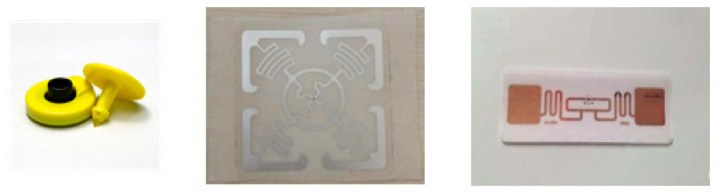
The UHF RFID tag.

The system is practically applied to a small farm where livestock wear RFID electronic ear tags during the breeding stage and basic information is entered into the ear tags. The reader collects the relevant information from the ear tag and uploads it to the breeding base data server through the computer. The RFID tags used are shown in Figure 17. In the slaughtering stage, the RFID ear tags from the breeding stage are converted into paper tags, and for the qualified products, the information from the bound RFID tags is uploaded to the database of the traceability system platform. In the circulation stage, the automatic recording logistics status information, including logistics company information and product transit-related information, is possible with the use of RFID technology. The selling stage can convert RFID tags into QR codes or bar codes to provide consumers and upload the relevant information to the platform data center, and end users can query all information of meat product information through the platform.

**Figure 18:**
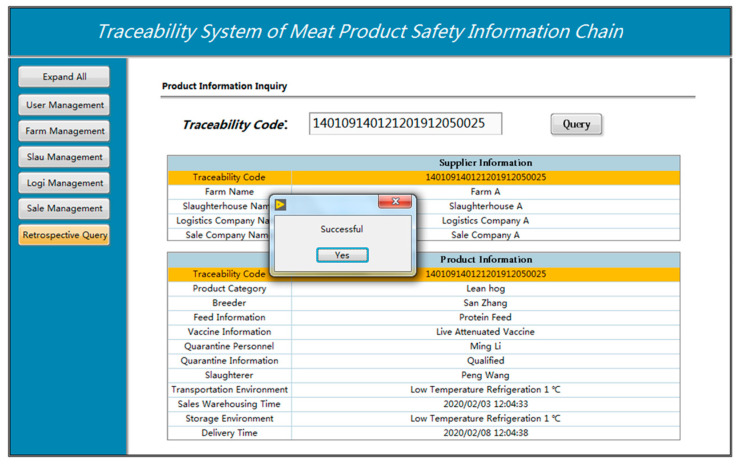
Traceability platform testing.

As shown in the Figure 18, in order to complete the information inquiry function for the supplier information and product information of the product, click the traceability inquiry button on the left side of the screen after logging into the traceability system and after entering the traceability code on the right side, such as 140109140121201912050025 in this design.

A traceability label for livestock and poultry meat products, which includes a traceability code, will be given to the operator or the final consumer in the trade of those items. Customers can scan the barcode or QR code on the package at supermarkets or convenience stores to view the traceability information of the meat items they have purchased on their smartphones. Additionally, you can view the product information by using the integrated device located at the sales terminal. Consumers can receive the desired traceability information by inputting the traceability code. The name of the farm, the name of the slaughterhouse, the name of the logistics company, the name of the sales company, and more specific information specific to the production and circulation of meat products, such as product type, breeder, feed information, vaccine information, quarantine personnel, quarantine information, slaughter personnel, logistics, and transportation are among the information that can be queried by this traceability query module. The supply chain information for meat products can be very useful for traceability.

### 3.3. Anti-Collision Algorithm Research and Improvement

In Pure Aloha (PA) algorithm, the label makes an answer to the question and answer signal of the reader. If there is a tag collision, the tag will wait for an arbitrary amount of time before responding to the reader once more. As a result of the relatively small amount of data transmitted, the short amount of time spent, and the fact that read-only tags send their data again after some time after colliding with another tag, the PA algorithm is essentially only applicable to read-only tags [26]. However, the amount of data transmitted by the tags when they are being read and written is relatively large, raising the probability of a collision.

The Slotted-ALOHA (S-ALOHA) algorithm has been proposed on the basis of the PA algorithm [27]. In the S-ALOHA algorithm, the PA algorithm idea is retained and time control is added. During the system identification, the tag transmits data at the beginning of the time slot. Time slots allow tags to only communicate with readers during defined times, resolving the collision issue with the PA algorithm. The communication process between the reader and the label is carried out over a channel that is divided into several time slots of equal size. These time slots must match the amount of time required for the reader and the label to exchange data; if they are less than the required amount of time, it will possibly produce the communication abnormality; if they are longer than the time, the channel will become idle and the reader will wait, reducing the performance of the algorithm.

The Q algorithm used in this design is a Dynamic Frame Slotted ALOHA (DFSA) algorithm to solve this problem because the above-mentioned algorithm is inefficient. To resolve the problem of the recognition efficiency being suddenly high and low due to different label quantities, the frame size will be dynamically adjusted. If the reader cannot identify a label in a frame because the label has collided, the number of time slots should be increased, and the size of the frame should be adjusted until the reader can identify the label. When a tag is recognized, the number of time slots and the value of frames are both decreased, resulting in a total number of tags and time slots that is close to being equal. At this point, the system has reached its maximum efficiency. According to the experimental analysis above, the system throughput rate can reach 36.7%.

## 4. Conclusions

In this paper, UHF RFID-based meat safety information chain traceability system is developed. The primary improvement of this system is that it has a complete traceability information chain. It enables the accurate collection of critical information for different batches at different stages, including breeding, slaughtering, logistics and transportation, and selling solves the problem of an incomplete traceability system. The system designs a low-cost and practical UHF RFID reader. It uses the Q-value algorithm to effectively collect traceability information on meat products that are in production and circulation, and is compatible with LoRa wireless, USB serial port, RS485, and RJ45 Ethernet communication to identify tag information for wider application. LabVIEW was used in the design of the traceability system platform, which includes user management, login user management, farm management, slaughter management, logistics management, and sales management. Users can more easily and conveniently ask about traceability. The research work of this paper can serve as a reference for the establishment of other food traceability systems, such as those for vegetables.

## Data Availability

Not applicable.
